# Beyond Boundaries of a Trial: Post-Market Clinical Follow-Up of SOYA Patients

**DOI:** 10.3390/jcm13216308

**Published:** 2024-10-22

**Authors:** Carlos Gavín, Victoria Sebastián, Mercedes Gimeno, Pilar Coronel

**Affiliations:** 1 Hospital Universitario Fundación Alcorcón, 28922 Madrid, Spain; 2 Scientific Depart, mentMeiji Pharma Spain, 28802 Madrid, Spain

**Keywords:** osteoarthritis, hyaluronic acid, viscosupplementation, real-world evidence, traumatology

## Abstract

**Background/Objectives:** osteoarthritis (OA) is a leading cause of disability. With an aging population and rising obesity rates, OA presents a growing challenge to health systems worldwide. Current OA treatments involve a mix of pharmacological and nonpharmacological interventions. Viscosupplementation with hyaluronic acid (HA) has proven effective, especially in knee OA, leading to its recommendation in international guidelines. This study investigates the sustained benefits of a single intra-articular HA injection beyond one year in patients coming from the SOYA trial, considering the EU MDR 2017/745 emphasis on post-market follow-up. **Methods:** A prospective, observational, open, post-marketing study in a cohort of patients that participated in the SOYA trial. Follow-up was carried out by means of a telephone survey, and the data were anonymized and coded so that patients could not be identified. The study was approved by the Alcorcón Hospital Institutional Review Board (Alcorcón, Madrid, Spain). **Results:** In the follow-up of the SOYA trial, 81.5% of patients sustained positive effects for over 6 months after the trial ended. This correlated with improved daily functioning, enhanced mood, and high patient satisfaction. Younger age and milder OA grades were associated with prolonged treatment effects. Notably, 82% of patients with >6 months of improvement did not require additional medication. **Conclusions:** the results of this study support the safety and performance of Adant^®^ Plus as a treatment for patients with mild and moderate knee OA, with results lasting more than one year. Post-marketing studies are particularly relevant to examine the experience gained with the use of the device in routine clinical practice.

## 1. Introduction

Osteoarthritis (OA) is a prevalent degenerative joint disease characterized by the progressive deterioration of articular cartilage, subchondral bone alterations, and synovial inflammation [1]. It is a disabling condition ranked as one of the highest contributors to global disability [2,3], significantly affecting quality of life. Epidemiological studies indicate that the prevalence of OA is around 15% of the world population over 30 years of age, with higher rates in women and increasing with age [4]. The etiopathogenesis of knee OA is multifactorial, involving a combination of intrinsic and extrinsic factors. Age-related changes, genetic predisposition, obesity, joint injuries, and repetitive mechanical stress, among other factors, contribute to the disease [5]. The degradation of cartilage, which is a crucial component in joint function due to its mechanical and frictional properties [6], together with the formation of osteophytes [7], is critical in the development and progression of the disease. These pathological changes lead to a cycle of pain, reduced function, and further joint damage. With the aging of the population and the increase in obesity, it is expected that the burden of OA will become a major problem for health systems across the world [2,3].

Due to the chronic nature of the disease, the multiple factors involved, and the high frequency of associated comorbidities, the ideal treatment for OA is yet to be identified. Currently, a combination of pharmacological and nonpharmacological interventions is used, which varies depending on the grade of the disease and the patient’s characteristics [1,8,9]. Non-pharmacological approaches, such as weight management, physical therapy, and lifestyle modifications, are often first-line interventions. Pharmacological treatments include analgesics, nonsteroidal anti-inflammatory drugs (NSAIDs), and corticosteroid injections. However, these options may have limited efficacy and potential side effects [10].

Viscosupplementation with intra-articular injections of hyaluronic acid (HA) as a treatment for OA started at the end of the 20th century, with the aim of restoring the altered synovial fluid [11]. HA is a naturally occurring glycosaminoglycan found in synovial fluid, providing lubrication and shock absorption in the joint [12]. The rationale behind HA injections is to replenish the viscoelastic properties of the synovial fluid, reducing pain and improving joint function, although it has also been demonstrated to have other mechanisms by which it contributes to the restoration of joint function [13]. The efficacy of HA treatment in knee OA has been confirmed in several meta-analyses [14,15,16,17,18,19,20,21,22,23], concluding that HA is a valuable treatment option that provides important reduction in pain and improvement in function, especially in adequately selected patients [24]. Furthermore, clinical studies have demonstrated that HA can provide symptomatic relief, offering longer-lasting effects [25]. This has made HA an attractive option for managing knee OA. As a consequence, viscosupplementation is recommended in most of the international guidelines issued by Scientific Societies [26].

Treatment with HA traditionally consisted of several intra-articular injections administered at weekly intervals. However, there is an increasing demand for short treatments, resulting in fewer physician visits, less risk of adverse events, and lower costs for both patients and the healthcare system. Adant^®^ Plus is a formulation of sodium hyaluronate consisting of a 2% wt/vol solution of medium MW (1000 kDa) [27] HA obtained by fermentation, dosed in syringes containing 98 mg of HA. A clinical trial with this product with good results up to 12 months follow-up after a single injection (the SOYA trial) was already published [11].

From a regulatory point of view, the new European Medical Device Regulation [28], which entered into force in May 2021, applies to all medical device manufacturers who want to introduce their products to the EU market. This regulation sets high standards of quality and safety and requires clinical investigations for medical devices; in particular, it establishes the need to perform a post-market follow-up as part of the manufacturer’s quality management system during the whole life cycle of the product.

The aim of this clinical follow-up is to evaluate the long-term effectiveness of a single injection of intra-articular HA 6 months in patients with OA beyond their participation in the SOYA trial [11]. In addition, the new regulatory framework for medical devices established by EU Regulation 2017/745 on medical devices encourages the follow-up of the efficacy and safety of a medical device throughout its life cycle.

## 2. Materials and Methods

### 2.1. Participants and Study Design

This extension of the SOYA trial was approved by the Alcorcón Hospital Institutional Review Board (Alcorcón, Madrid, Spain) (meeting 26 January 2022; approval number 18/76). All patients participating in the study gave their written informed consent before being included. This study complies with the Declaration of Helsinki.

In the previously conducted study (SOYA trial), patients with OA grade 2–3 of Kellgren & Lawrence (KL) received a single intra-articular injection of the study product and were followed for one year. At 6 months, patients were offered a second injection if necessary. The primary endpoint was the reduction in pain at 12 months, measured with the visual analog scale (VAS) and the minimally clinical important improvement (MCII), defined as a relative improvement in pain ≥20% over baseline [11]. The present study, a follow-up after the SOYA trial ended, is a prospective, observational, open study in a cohort of patients who participated in this trial. The patients selected were those included in one of the participating centers, the Hospital Universitario Fundación Alcorcón (HUFA), as this was the hospital that recruited the largest number of subjects in the trial, and none of the patients was given the booster injection permitted in the SOYA protocol. This allows assessment of the duration of the effect of a single injection beyond the end of the SOYA trial in the usual clinical practice. As the patients came from the SOYA study, there were no additional inclusion/exclusion criteria for the study.

Follow-up was carried out 6 months after the end of the SOYA trial by means of a telephone survey due to the COVID-19 pandemic. A survey consisting of easy questions was used to determine the duration of the effects of Adant^®^ Plus 6 months after patients completed their participation in the SOYA trial. This survey contains questions related to quality of life and grade of satisfaction with the treatment (Table 1). The person who managed the questionnaire was the same for all patients and different from the investigator who administered the intra-articular treatment in the trial to avoid the possibility of bias in patient response. The data were anonymized and coded so that patients could not be identified.

### 2.2. Statistical Analysis

Qualitative variables were described using absolute frequencies and percentages. Quantitative variables that fitted to a normal distribution were described by mean, standard deviation (SD), minimum (Min), and maximum (Max), while those that did not fit to a normal distribution were described by median, interquartile range (first quartile [Q1]—third quartile [Q3]), Min, and Max.

Univariate comparisons between categorical variables were performed using the chi-square test and/or Fisher’s exact test. For continuous variables, the shape of the distributions was analyzed using the Kolmogorov–Smirnoff and Shapiro–Wilk tests. Comparisons between two unrelated means were made using Student’s *t*-test or the Mann–Whitney U test. In the case of analyzing more than two groups, ANOVA tests or the Kruskal–Wallis test were used for comparisons.

The statistical analysis was performed with SAS^®^ v9.3, and significance was set at *p* < 0.05.

## 3. Results

**Figure 1:**
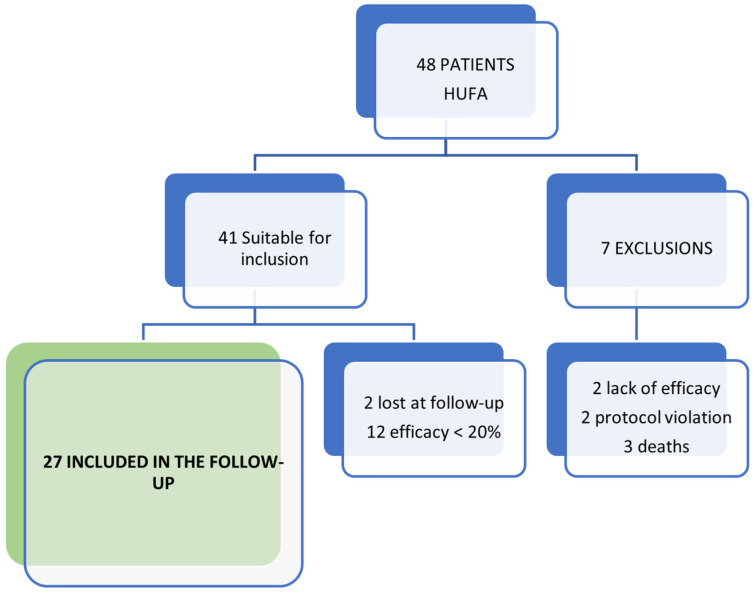
Scheme of patients included in the follow-up of the SOYA trial.

Of the 48 patients included in the SOYA trial at HUFA, those who did not complete the trial were excluded (2 due to lack of efficacy and 2 due to protocol violation). Also excluded were those patients who did not reach the MCII at the end of the trial (*n* = 12). In addition, three patients had died at the moment of completing the questionnaire, and two patients could not be contacted. The final follow-up sample consisted of 27 patients, as shown in Figure 1.

The questionnaire was run between February and March 2022. The mean age of the population included in the follow-up was 69 years; the majority were women (67%), and the body mass index was 26.4 kg/m^2^. Almost half of them (48%) had an active working life. The Kellgren grade was almost equally distributed between grades 2 and 3. More than half of the sample (55.6%) were taking at least one drug at the end of the SOYA trial (Table 2).

**Figure 2:**
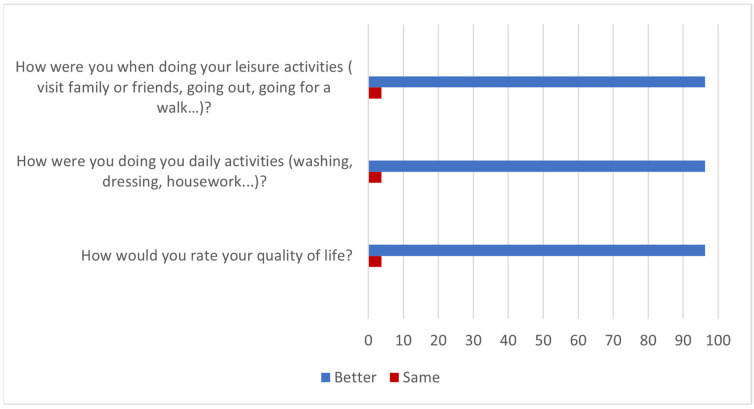
Improvement in quality of life over the duration of treatment effects (% of patients).

According to the results of the questionnaire, the effects of the treatment with HA were maintained for more than 6 months in 81.5% of patients after completion of the trial (Question 1). The 96% said they felt better in their daily and leisure activities (Questions 2 and 3) while the improvement lasted and, therefore, had a better quality of life (Question 4). These results are summarized in Figure 2.

In accordance with the above results, the majority of the sample (96%) reported being in a better mood thanks to the treatment (Question 5).

**Figure 3:**
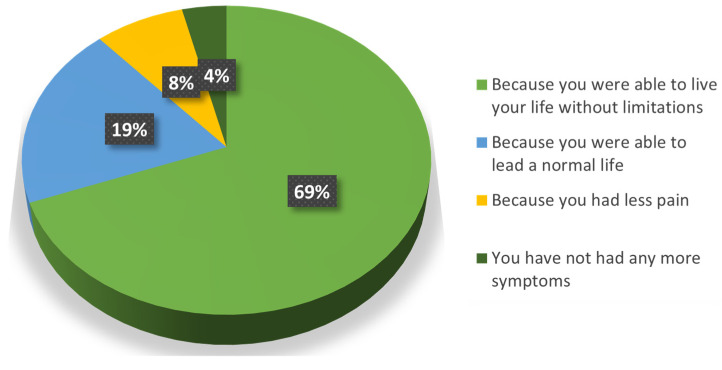
Reasons why patients were satisfied with the treatment.

Almost the whole population (96%) was satisfied with the treatment (Question 6a) because they could lead a normal life without limitations (88%) (Question 6b) (Figure 3). Only one patient was dissatisfied because the treatment “did not change their symptoms”, and this perception is reflected in the majority of his responses. Among all the follow-up patients, this was the one with the smaller reduction in pain at the end of the SOYA study.

All the patients would recommend the treatment (Question 7a) because they experienced a significant reduction in pain (96%), which, in 78% of the sample, completely disappeared (Question 7b).

**Figure 4:**
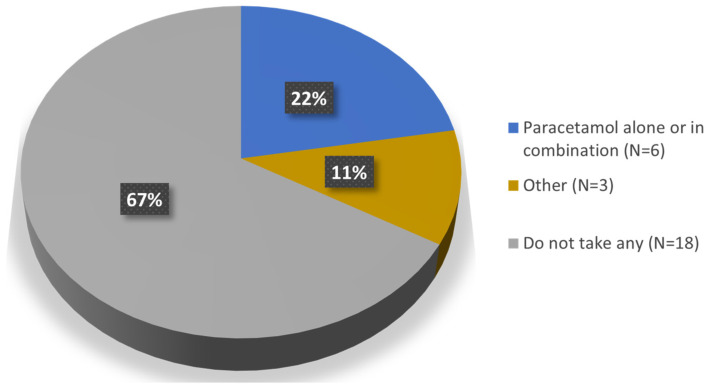
Concomitant medication for the injected knee.

Regarding concomitant medication (Question 8), 67% of the patients (*n* = 18) reported not taking any medication for the injected knee; the remaining 33% (*n* = 9) were taking some medication which, in six of the patients consisted of paracetamol alone or in combination; of these six, three patients take it occasionally (Figure 4).

**Figure 5:**
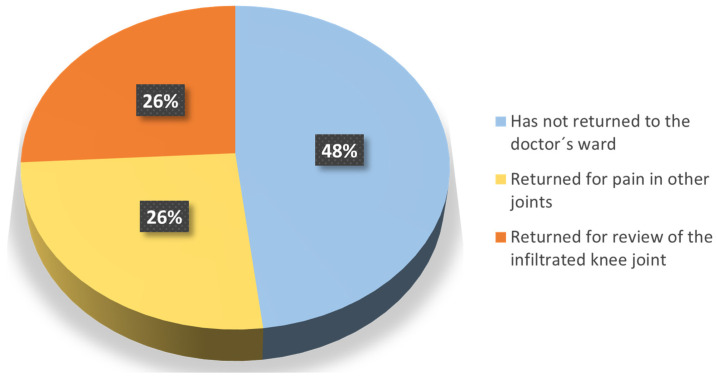
Reasons for returning to the doctor’s ward.

The patients were also asked about the medical follow-up of their OA. At the time when the questionnaire was made, 74% had not returned to the doctor’s ward or returned but for pain in other joints, while the remaining 26% returned to the clinic due to the injected knee (Question 9) (Figure 5). In addition, the vast majority (96%) confirmed that they were satisfied with the follow-up of their OA after the end of the trial (Question 10a), and 26% considered HA as a possible option to delay surgery (Question 10b).

**Figure 6:**
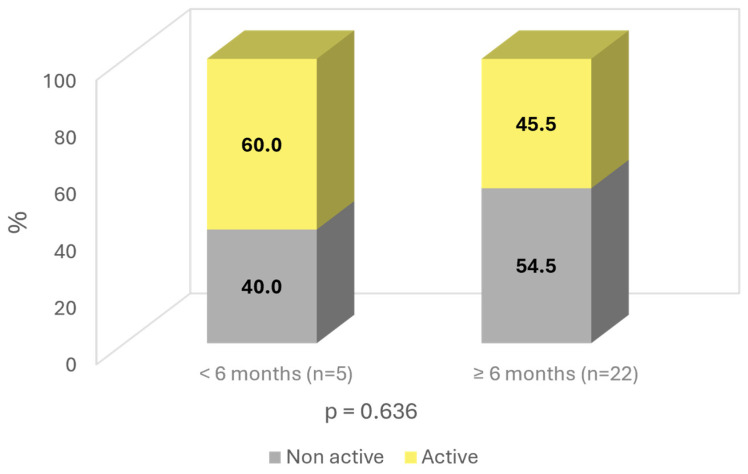
Time of improvement by activity.

The patients were also analyzed based on the duration of the effects after the end of SOYA. More than 6 months—81.5% (*n* = 22); less than 6 months—18.5% (*n* = 5). Age was found to influence the duration of the improvement, with younger patients having a longer duration of effects, with a *p*-value almost reaching statistical significance (*p* = 0.086). Similarly, a longer duration of effects was observed in “non-active” patients, with a difference of 9% compared to “active working” subjects (54.5% vs. 45.5%; ns) (Figure 6).

**Figure 7:**
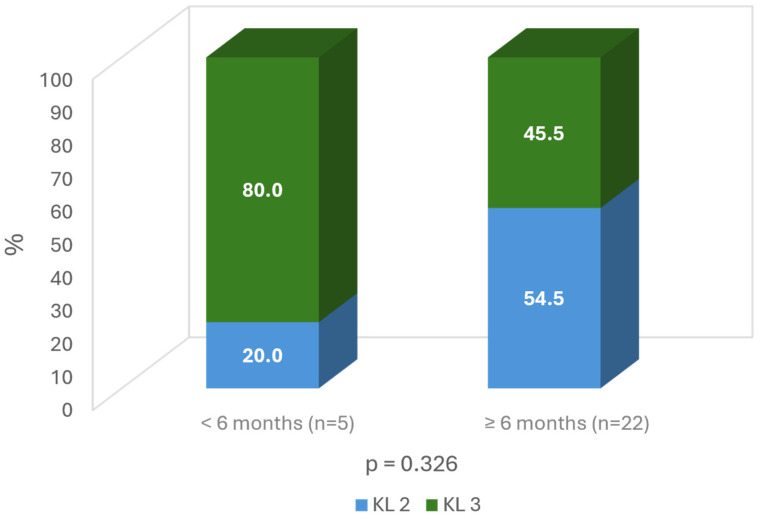
Time of improvement according to Kellgren grade on the target knee.

When considering the severity of the OA, the milder OA grade, the longer duration of improvement (more than 6 months: 54.5% grade 2 vs. 45.5% grade 3; ns) (Figure 7).

**Figure 8:**
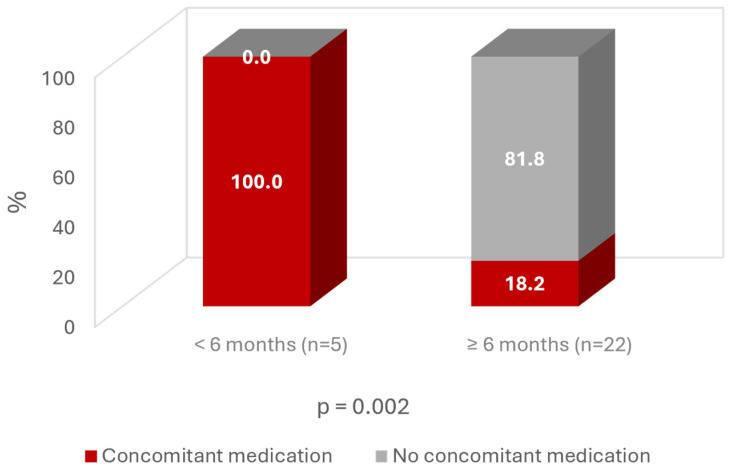
Time of improvement according to concomitant medication use.

Regarding concomitant medication, all patients with an improvement <6 months were taking other medication during follow-up. In contrast, almost 82% of patients with an improvement time >6 months were not taking any medication (Figure 8).

## 4. Discussion

Currently most clinical guidelines provide recommendations about intra-articular treatments for OA treatment. A recent systematic review concluded that HA and corticosteroids (CS) are recommended in most of them [26], while PRP or other biological therapies still have insufficient evidence to make a recommendation for or against its use [26,29].

The care of patients with OA is initiated with non-pharmacological measures and oral options. When oral drugs fail or are contraindicated, intra-articular treatments are usually recommended. For faster onset but shorter-term symptom relief, IA-CS injection is generally recommended. For a delayed but longer-term symptom relief period, IA-HA should be considered. The duration of the effects after intra-articular treatments is also a controversial matter, and even published meta-analyses achieve conclusions that, at first glance, seem conflicting. The MA from Jevsevaar concluded that CS had a larger effect than HA [30], whereas the MA from Bannuru found that HA had the largest treatment effect of knee OA treatments analyzed [31]. However, these studies have an underlying limitation associated with the short follow-up period conducted in the trials. In general, there are scarce studies with long follow-ups (>6 months), limiting the conclusions that can be drawn about the duration of the effect. The exception is a limited number of studies that were designed with a long follow-up, such as the AMELIA project [25], where the treatment with several cycles of multiple injections showed a carry-over effect.

The main objective of the present work was to evaluate the long-term effects in a cohort of patients who participated in the SOYA trial. Only patients that reached the MCII at the end of the trial were considered. The additional follow-up was carried out by means of a telephone survey using a questionnaire with easy questions, and no inconsistencies were observed in the answers provided by the patients. Half of the patients consulted were active, the majority were women, and OA grades 2–3 were equally distributed.

The survey showed that after the end of the study, the efficacy of the treatment with HA was maintained for more than 6 additional months in 81.5% of the patients (that means at least 18 months), with a significant reduction in pain in 78% of the sample considered had disappeared. As a result, patients felt better, which translated into a better mood and better quality of life. At the time when the questionnaire was made, 74% had not returned to the doctor’s ward or returned but for pain in other joints.

Younger patients, with a lower degree of OA in the injected knee and with lower pain score at the end of the SOYA trial, are the ones who presented a longer duration of improvement, extending their effects to >6 months; although, due to the sample size, statistical significance was not reached.

Regarding concomitant medication, 67% of the patients reported not taking any medication for the injected knee; the remaining 33% were taking analgesics, mainly occasional paracetamol. It is interesting to highlight that almost 82% of patients with a sustained improvement >6 months were not taking any medication for OA. This is a condition to be taken into account considering the direct costs of analgesics and the indirect costs related to associated risks common among elderly people [32,33], as well as the current epidemics of opioid abuse [34,35].

The prolonged duration of the effects after a single injection in a significant number of patients is remarkable and would have gone unnoticed if this follow-up had not been performed. These patients were those who already had a good response at the end of the SOYA trial, so this study highlights the importance of clinical follow-up after clinical trials and once the product is introduced in clinical practice. In addition, the importance of good patient selection when prescribing any treatment should be emphasized in order to achieve optimal results [24,36].

The vast majority (96%) of our patients confirmed that they were satisfied with the follow-up of their OA at the hospital after the end of the trial, and 26% considered HA as a possible option to delay surgery. This is of interest considering that they had received a single injection, and it has been demonstrated that repeated administration maintains or further improves the effects without increasing the risk and is associated with the delay of knee replacement surgery for up to 3 years [37,38,39,40].

One of the limitations of this study is the small sample size, and the ideal condition would have been to perform it with all patients who participated in the trial. However, the sample considered represents almost 50% of the study patients and is homogeneous, having been recruited and treated in a single hospital. Another limitation was the need to follow-up by telephone, as in the early post-pandemic period, when the questionnaire was administered, face-to-face consultations at the hospital were still limited. A controlled study with a larger sample size would be useful to further assess the long-term efficacy of a single HA injection and to support our findings.

Situations detected in the past, such as the risks arising from metal-on-metal hip prostheses or the scandal of defective “PIP” silicone breast implants, have resulted in the new regulation governing medical devices, which has, among its main objectives, the improvement of patient safety through stricter assessment procedures as well as post-market clinical follow-up once the products have been authorized.

## 5. Conclusions

The results shown here provide a rationale for the use of Adant^®^ Plus as a treatment for patients with mild and moderate knee OA, with results lasting for more than one year after a single injection in a significant number of patients. Furthermore, this study highlights the worth of carrying out long-term clinical follow-ups after the trial ends, with this evaluation being crucial to understanding real-world performance and safety.

## Figures and Tables

**Table 1:** SOYA study patient follow-up questionnaire.

1 After our last contact about your participation in the hyaluronic acid clinical trial, how long do you estimate that the effects of the injection lasted?
2 While the effects lasted, how were you doing your daily activities (washing, dressing, housework)?
3 While the effects lasted, how did you feel about your leisure activities (visiting family or friends, going out, going for a walk…)?
4 How would you rate your quality of life while the effects lasted?
5 Was your mood better because of the treatment?
6 (a) Overall, were you satisfied with the treatment? (b) Why?
7 (a) Would you recommend this treatment to someone with the same symptoms of osteoarthritis as you? (b) Why?
8 Are you currently taking any medication for the knee that was injected? If yes, please describe it briefly.
9 Why haven’t you returned to the doctor’s ward since your participation in the study ended?
10 (a) In general, are you satisfied with the medical follow-up of your osteoarthritis? (b) Why?

**Table 2:** Characteristics of patients included in the follow-up (*n* = 27).

Variable	Included *n* = 27
Sex female (*n*, [%])	18 (66.7%)
Age, mean (sd)	69.0 (9.3)
Body mass index (BMI), mean (sd)	26.4 (3.0)
Active working life (*n* [%])	13 (48.1%)
Kellgren–Lawrence grade 2/3 (*n* [%])	13 (48.1%)/14 (51.9%)
Pain in VAS at end of SOYA trial, mean (sd)	19.1 (14.6)
Taking at least 1 drug (*n* [%])	15 (55.6%)

**Abbreviations:** VAS, visual analog scale; SOYA, symptomatic osteoarthritis one-year assessment.

## Data Availability

The original contributions presented in the study are included in the article, further inquiries can be directed to the corresponding author.
